# The Multi-Omic Prognostic Model of Oxidative Stress-Related Genes in Acute Myeloid Leukemia

**DOI:** 10.3389/fgene.2021.722064

**Published:** 2021-09-30

**Authors:** Chao Dong, Naijin Zhang, Lijun Zhang

**Affiliations:** ^1^Department of Hematology, The First Affiliated Hospital of China Medical University, Shenyang, China; ^2^Department of Cardiology, The First Affiliated Hospital of China Medical University, Shenyang, China

**Keywords:** acute myeloid leukemia, multi-omics, oxidative stress, prognosis, immunitary

## Abstract

**Background:** Acute myeloid leukemia (AML) is one of the most common cancers in the world, and oxidative stress is closely related to leukemia. A lot of effort has been made to improve the prognosis of AML. However, the situation remains serious. Hence, we focused on the study of prognostic genes in AML.

**Materials and Methods:** Prognostic oxidative stress genes were screened out. The gene expression profile of AML patients was downloaded from the The Cancer Genome Atlas (TCGA) database. The oxidative stress-related model was constructed, by which the prognosis of AML patients was predicted using the two GEO GSE23143 datasets and the stability of the GSE71014 authentication model.

**Results:** The prognostic oxidative stress genes were screened out in AML, and the prognostic genes were significantly enriched in a large number of pathways based on Gene Ontology (GO) and Kyoto Encyclopedia of Genes and Genomes (KEGG) enrichment analysis. There was a complex interaction between prognostic genes and transcription factors. After constructing the prediction model, the clinical predictive value of the model was discussed in a multi-omic study. We investigated the sensitivity of risk score to common chemotherapeutic agents, the influence of signaling pathways on the prognosis of AML patients, and the correlation of multiple genes with immune score and immune dysfunction.

**Conclusions:** A highly effective prognostic risk model for AML patients was established and validated. The association of prognostic oxidative stress genes with drug sensitivity, signaling pathways, and immune infiltration was explored. The results suggested that oxidative stress genes promised to be potential prognostic biomarkers for AML, which may provide a new basis for disease management.

## Introduction

Acute myelogenous leukemia (AML), the most common acute leukemia in adults, is a group of clonal malignant proliferative diseases caused by acquired somatic cell genetic damage and the accumulation of hematopoietic progenitor cells, which is highly heterogeneous (Pelcovits and Niroula, 2020). The prognosis of AML is extremely unstable with a low survival rate, with less than 50% 5-year overall survival (OS) rate in younger AML patients, and only 20% 2-year OS rate after diagnosis in older patients, and there are only about 10% AML patients over 60 years of age (Estey, 2018; Shallis et al., 2019). Most patients do not have any detectable early symptoms, and they usually present with acute complications of bone marrow failure (Abelson et al., 2018). At present, chemotherapy remains the main treatment for AML. However, 70% of patients who achieve remission will eventually suffer from relapse and disease evolution into refractory leukemia, resulting in death due to treatment failure. AML patients show different outcomes after chemotherapy, and molecular abnormalities and cytogenetic characteristics at diagnosis are considered as the most critical prognostic parameters (Chen X. X. et al., 2020). The etiology and pathogenesis of leukemia are very complex. Despite the progress in the pathogenesis of AML and the continuous emergence of new drugs, the prognosis of AML is still poor (Estey, 2018). The development of new therapies, combined with the improvement of genetic profiling and risk stratification, is expected to lead to incremental gains in remission and survival (De Kouchkovsky and Abdul-Hay, 2016). Currently, it is believed that the vast majority of AML is caused by the interaction between environmental factors and cell genetic materials, and the level of reactive oxygen species (ROS) may act as an influencing factor.

A variety of ROS produced in the human body has been identified as by-products of cellular aerobic metabolism, persistent stress, and exposure to ultraviolet or X-ray rays. ROS also plays a crucial role in the regulation of cell signaling and cytokines, growth factors and hormones, transcription, ion transport, neuro-regulation, immune regulation, and apoptosis (Gupta et al., 2014). Oxidative stress, as a physiological disorder, is caused by the imbalance between the free radicals (produced in the process of oxidation) and the ability of the body to remove them under some pathological factors. A previous study has shown that ROS levels in leukemia cells is higher than that in normal cells (Klaunig, 2018). The fact that ROS levels in cancer cells is higher than that in normal cells could serve as a new way to target tumor cells. The changes of REDOX are directly related to the process of proliferation, anti-apoptosis, and drug resistance of leukemia cells. Therefore, regulating ROS production and the expression of antioxidant oncogenes can affect the apoptotic pathway and then ultimately control leukemia progression (Samimi et al., 2018). Targeting REDOX-sensitive pathways and transcription factors holds great promise for cancer prevention and treatment. Various drugs have been found to be able to interfere with REDOX cell signaling pathways (Reuter et al., 2010). There is an urgent need to develop biomarkers for predicting the response to prooxidative therapy, with the purpose to determine the best option for cancer eradication. The multi-omics research method may contribute to understanding the underlying pathophysiological processes associated with this disease more comprehensively (Sosa et al., 2013; Testa et al., 2016; Prada-Arismendy et al., 2017).

Therefore, it is of great significance to establish a risk prediction model for AML management by screening AML prognosis-related genes. TCGA database, which is a project jointly supervised by the U.S. National Cancer Institute, Human Genome Research Institute, and the biggest cancer gene information database, has not only comprehensively reflected on the type of carcinoma, but also showed on multiple omics data (such as gene expression data, the miRNA expression data, copy number variation, DNA methylation, and SNP). In the early stage, oxidative stress-related genes were screened through the oxidative stress gene set; oxidative stress-related models were constructed through the TCGA data set, with its stability verified in two GEO data sets, by which the prognosis of AML patients was predicted. In summary, this study may contribute to the prognosis or monitoring of AML, as well as to clinical therapeutic decision-making, which may provide new evidence for the management of the disease.

## Materials and Methods

### Data Acquisition

The TCGA database^1^, the biggest cancer gene information database, contains data of gene expression, miRNA expression and copy number variation, DNA methylation, SNPs, and other data. The original mRNA expression data of processed AML were downloaded, and the data expression profiles of 151 patient samples were obtained. The Series Matrix File data of GSE23143 were downloaded from the NCBI GEO public database; the annotation platform was GPL570. Data of 86 AML patients with complete profiles of expression and survival information were extracted. The Series Matrix File data of GSE71014 were downloaded; the annotation platform was GPL10588. Data of 104 AML patients with complete profile of expression and survival information were downloaded. Moreover, 259 oxidative stress-related genes were obtained through Amigo^2^.

### Gene Ontology and Kyoto Encyclopedia of Genes and Genomes Functions

ClusterProfiler (R3.6) was used to annotate the functional factors of differences (Yu et al., 2012) to comprehensively explore the functional correlation of these prognostic genes. GO and KEGG were used to assess the related functional categories. For the analysis of the GO and KEGG enrichment pathways, *P* and *Q* values less than 0.05 were considered as significant categories.

### Model Construction and Prognosis

Oxidative stress-related genes were selected. Lasso regression was used to construct the prognostic model. After the inclusion of expression values for each specific gene, a risk score formula for each patient was constructed and weighted based on its estimated regression coefficient value in Lasso regression analysis (Tibshirani, 1996; Sauerbrei et al., 2007). According to the risk scoring formula, patients were allocated into the low-risk group and high-risk group, with the median risk score as the cut-off point. Survival differences between the two groups were assessed by Kaplan–Meier (KM) and compared using log-rank statistical methods. Lasso regression analysis and stratified analysis were used to examine the role of the risk score in the prediction of patient outcomes. The accuracy of model prediction was analyzed using the ROC curve.

### Drug Sensitivity Analysis

The Genomics of Drug Sensitivity in Cancer (GDSC) database^3^ and R software package “pRRophetic” were used to predict the chemotherapy sensitivity of each tumor sample. The estimated IC50 value of each specific chemotherapeutic agent was obtained by the regression method. A total of 10 cross-validations were performed with the GDSC training set to test the regression and prediction accuracy (Geeleher et al., 2014). Default values were selected for all parameters, including “combat,” which removes the batching effect and the average of duplicate gene expression.

### Analysis of Immune Cell Infiltration

The CIBERSORT algorithm was used to analyze the RNA-seq data of AML patients in different subgroups (Chen et al., 2018) to infer the relative proportion of 22 types of immuno-infiltrating cells. Spearman correlation analysis was performed on gene expression and immuno-cell content. *P* < 0.05 was considered to be statistically significant.

### Gene Set Enrichment Analysis

The expression profiles of AML patients were analyzed through GSEA^4^ to identify the differentially expressed genes in patients in the high-risk and low-risk groups (Subramanian et al., 2005). The gene set was filtered using a maximum (500) and minimum (Tibshirani, 1996) gene set. After 1,000 permutations, a rich gene set was obtained based on *P* < 0.05. Finally, the significantly enriched genes in GO and KEGG were centrally displayed.

### Tumor-Immune System Interactions Database Analysis

As an online website for the interaction of tumor and immune system (Ru et al., 2019), tumor-immune system interactions database (TISIDB) integrates multiple heterogeneous data types. The data were then combined into ten categories of information for each gene. TISIDB integrates data from multiple databases (such as TCGA, UNIPROT, GO, and DRUGBANK), which is a valuable resource for cancer immunology research and treatment. Relevant data were downloaded from the TISIDB website^5^ to study the interaction between tumor and immune system.

### Tumor Immune Dysfunction and Exclusion Analysis

The response of patients was predicted using the tumor immune dysfunction and exclusion (TIDE) module by estimating several published transcriptome biomarkers based on the tumor pre-treatment expression profiles. The immune dysfunction and rejection reaction were analyzed through TIDE official website^6^.

### Statistical Analysis

Survival curves were generated by the KM method and compared by log-rank. The Cox proportional risk model was used for multivariate analysis. All statistical analyses were conducted in R language (version 3.6). All statistical tests were bilateral, and *P* < 0.05 was indicative of a statistically significant difference.

### Weighted Gene Co-expression Network Analysis

Through the construction of the weighted gene co-expression network, the co-expression gene modules were identified, and the relationship between gene network and phenotype, as well as core genes in the network, were explored. The co-expression network of all genes in the data set was constructed using the WGCNA-R packet, and the top 5000 genes with variance were screened by this algorithm for further analysis, with the soft threshold of 7. The weighted adjacency matrix was transformed into the topological overlap matrix (TOM) to estimate the network connectivity, and the cluster tree structure of TOM matrix was constructed using the hierarchical clustering method. Different branches of the cluster tree represent different gene modules, and different colors represent different modules. According to the weighted correlation coefficient of genes, the genes were classified; the genes with similar patterns were grouped into one module, and tens of thousands of genes were divided into multiple modules through gene expression patterns.

## Results

### To Explore the Prognostic Oxidative Stress Genes in Acute Myeloid Leukemia

The original mRNA expression data (FPKM) of processed AML were downloaded from the TCGA database. Based on this expression profile, the expression profiles of 450 oxidative stress-related genes were extracted. After screening out some extremely low-expressed genes, the remaining genes were used as candidate gene sets for the subsequent modeling analysis. Moreover, the clinical information of AML patients was collected to further identify key genes in the concentration of oxidative stress genes. A combination of Cox univariate regression and Lasso regression feature selection algorithm was used to select the characteristic genes in AML. As shown by the results, 89 prognostic related genes (*P* < 0.05) were screened by Cox univariate regression.

### Subgroup Analysis Based on Prognostic Related Oxidative Stress Gene Clustering

ConsensusClusterPlus (Wilkerson and Hayes, 2010) was used to conduct a consistency cluster analysis on the expression of prognostic genes in the R package. This study found that AML patients could be allocated into three subgroups when the cluster number *K* = 3 (Supplementary Figure 1). The KM-plot survival analysis was further performed, and the results revealed that there were significant differences in survival among the three groups, with subgroup 1 showing the worst prognosis and subgroup 3 showing the best prognosis (Supplementary Figure 2).

### Functional Enrichment of Prognostic Genes and Construction of Transcriptional Regulatory Networks

**FIGURE 1:**
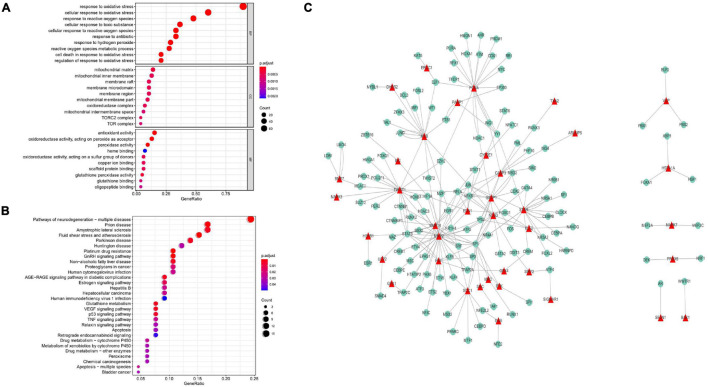
GO enrichment analysis of prognostic genes. **(A)** In the GO rich concentration, there were responses to oxidative stress, cellular response to oxidative stress, response to ROS, response to engineering, cellular response to ROS, and other pathways. KEGG enrichment analysis of prognostic genes. **(B)** In the process of KEGG enrichment, there were pathways of neurodegeneration, apoptosis, MAPK signaling pathway, and other Pathways. **(C)** Transcriptional interaction network analysis. Thirty-eight prognostic genes had complex interactions with transcription factors.

A total of 89 prognostic genes were enriched by GO and KEGG analysis, which were found significantly enriched in a large number of pathways. For example, in GO rich concentration, there were responses to oxidative stress, cellular response to oxidative stress, response to ROS, response to the antibiotic, and cellular response to ROS and other pathways (Figure 1A). Based on KEGG enrichment analysis, there were neurodegeneration and apoptosis-related pathways, MAPK signaling pathway, and other pathways (Figure 1B). The transcription factors that interact with the prognostic genes were further predicted through the Trusts transcription factor database, and the results showed that 38 prognostic genes had complex interactions with transcription factors. Finally, the interactions between these 38 prognostic genes and transcription factors were demonstrated through Cytoscape (Figure 1C).

### The Prognostic Related Genes Were Obtained and a Prediction Model Was Built

**FIGURE 2:**
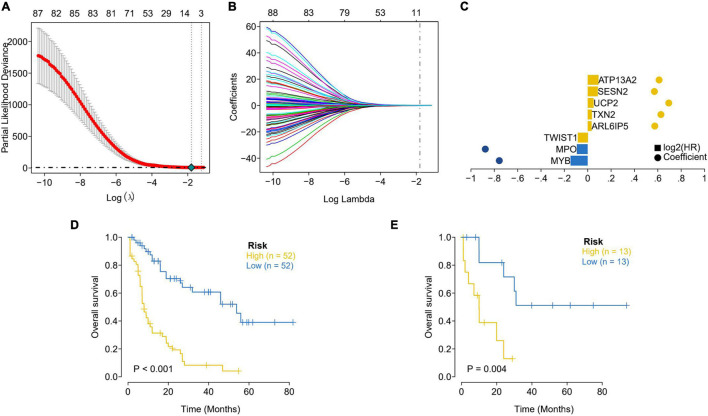
Lasso regression analysis was used to select the optimal coefficients. **(A)** Screening the best Partial Likelihood Deviance value and the best Lambda value. **(B)** The optimal coefficient of each gene was obtained through the optimal Lambda. **(C)** Display of LASSO regression results for each key gene. The distance between the bubbles represents the coefficients; the height of the column represents the size of the log2 (HR) value. **(D)** Assessment of survival differences between the high-risk (52 samples) and low-risk groups (52 samples) in the training dataset. **(E)** Internal validation focused on the assessment results of survival differences between the high-risk (13 samples) and low-risk (13 samples) group. The yellow line represents the high-risk group and the blue line represents the low-risk group.

**FIGURE 3:**
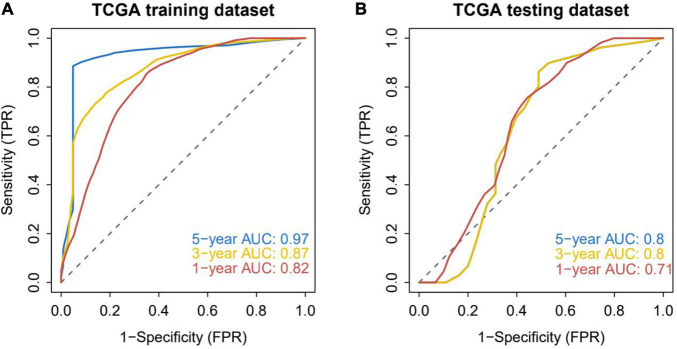
Accuracy analysis of model prediction. **(A)** ROC analysis of the accuracy of the training set model: the AUC values of 1, 3, and 5 years in the training dataset are all greater than 0.8, indicating that the model has good verification efficiency. **(B)** ROC analysis of the accuracy of the internal validation set model: the AUC values in the testing dataset for 1, 3, and 5 years are all greater than 0.7, indicating that the model has good validation efficiency.

TCGA patients were randomly allocated into the training set and validation set at 4:1 ratio. The best risk score (Risk Score = MYB × (−0.14587378) + MPO × (−0.092353084) + TWIST1 × (−0.084998334) + ARL6IP5 × 0.032888142 + TXN2 × 0.035097965 + UCP2 × 0.050187897 + SESN2 × 0.085314112 + ATP13A2 × 0.091438829) values, obtained by LASSO regression analysis, were used for the subsequent analysis. Patients were allocated into the high-risk and low-risk groups (based on the median risk score) and analyzed using KM curves. The OS of the high-risk group in both the training set and the test set was significantly lower than that of the low-risk group (Figure 2). Moreover, ROC curve results showed that the AUC values of 1, 3, and 5 years were all greater than 0.8 and 0.7 in the training set and the test set, respectively, indicating the good verification efficiency of the mode (Figure 3).

### The Clinical Predictive Value of the Model Was Discussed in Multi-Omic Study

“Omics” integrates biology, various technologies, and clinical observation. The use of “big data” methods to study human diseases has become a trend (Dunn and Meerzaman, 2020). AML, a common and fatal hematologic malignancy, is highly dependent on the bone marrow microenvironment. The tumor microenvironment (TME) is mainly composed of tumor-related fibroblasts, immune cells, extracellular matrix, growth factors, inflammatory factors, special physical and chemical characteristics, and cancer cells. The tumor microenvironment remarkably affects the diagnosis, survival outcome, and clinical treatment sensitivity of AML (Huang et al., 2019; Zhang et al., 2020).

By analyzing the relationship between risk score and tumor immuno-invasion, the potential molecular mechanism of risk score affecting AML progression was further explored. The results showed that risk score was positively correlated with monocytes, M2-type macrophages, and activated memory CD4 T cells, and negatively correlated with eosinophils, T cells gamma delta, and resting mast cell (Supplementary Figure 3A). Patients with early AML treated with chemotherapy have a clear response. Based on the drug sensitivity data from the GDSC database, our study predicted the chemotherapy sensitivity of each tumor sample by R package “pRRophetic,” and further explored the risk score and the sensitivity of common chemotherapy drugs. It was found that risk score significantly affected patients’ sensitivity to Bexarotene, Bortezomib, Bryostatin 1, Dasatinib, Metformin, and Paclitaxel (Supplementary Figure 3B). The genetic mutations of patients in the high-risk and low-risk groups were further analyzed, with the results presented in the mutation map (Supplementary Figure 3C).

A previous study has shown that Dasatinib not only inhibits the BCR-ABL kinase of chronic myeloid leukemia, but also inhibits KIT kinase, including KIT mutation encoded KIT kinase. At a follow-up of 4 years, Dasatinib had a longer 4-year EFS (event-free survival) (58% vs. 48%) (Estey, 2018).

### Discussion on the Specific Signaling Mechanisms Related to Prognostic Models

The specific signaling pathways involved in the high-risk correlation model were then investigated and the potential molecular mechanisms were explored, by which risk score influences tumor progression. Finally, through GSEA analysis, significant enrichment was found in many related pathways. Some of these highly significant pathways were separately shown in a concentrated manner (Supplementary Figure 4). GO results showed that patients in the high-risk group mainly enriched the TUMOR NECROSIS FACTOR MEDIATED SIGNALING PATHWAY, INTERFERON GAMMA PRODUCTION, and other SIGNALING pathways. According to KEGG results, the high-risk group mainly enriched the SIGNALING pathways such as the NATURAL KILLER CELL MEDIATED CYTOTOXICITY and Chemokine SIGNALING PATHWAY, which suggested that the disturbance of these SIGNALING pathways in the low-risk group affected the prognosis of AML patients.

### External Data Sets Were Used to Verify the Robustness of the Prognostic Model

**FIGURE 4:**
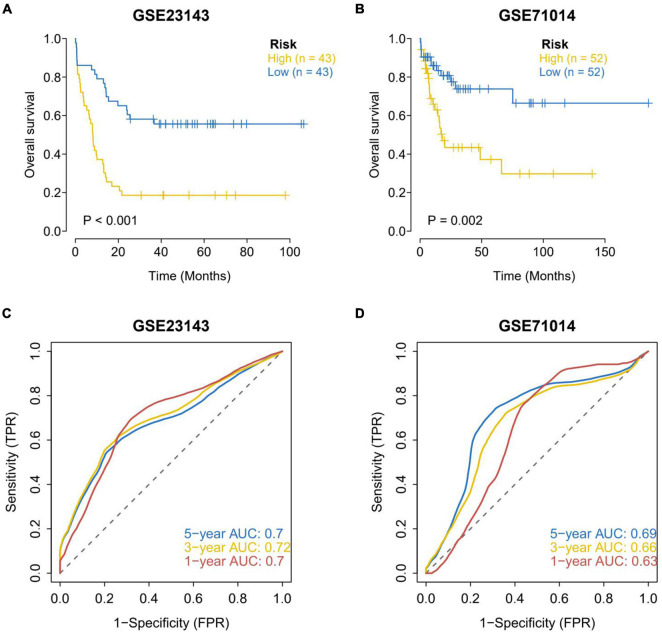
Assessment of survival differences between the high-risk and low-risk groups in the external validation set. **(A)** Assessment of survival differences in external validation set 1, in which 43 samples were assigned into the high-risk group and low-risk group, respectively. **(B)** External validation of the assessment of survival differences in external validation set 2, in which 52 samples were allocated into the high-risk group and the low-risk group, respectively. The yellow line represents the high-risk group and the blue line represents the low-risk group. The results showed that the OS in the high-risk group of the two GEO external validation sets was notably lower than that in the low-risk group. Accuracy analysis of external validation set model predictions. **(C)** ROC analysis of the accuracy of external validation set 1 model. **(D)** ROC analysis of the accuracy of the external validation set 2 model. AUC values of 1, 3, and 5 years in GSE23143 data set are not less than 0.7. AUC values of 1, 3, and 5 years in GSE71014 data set are all greater than 0.6. The results showed that the model has a strong predictive effect on the prognosis of patients.

The data of processed AML patients with survival data (GSE71014 and GSE23143) from the GEO database were downloaded. The clinical classification of AML patients in the GEO database was predicted according to the model. The survival difference between the two groups was evaluated by Kaplan–Meier, and the stability of the prediction model was investigated. The results showed that the OS in the high-risk group of the two GEO external validation sets was noticeably lower than that in the low-risk group (Figures 4A,B). ROC curve analysis was conducted to verify the accuracy of the model. It was found that the model had a strong predictive effect on the prognosis of patients (AUC values of 1, 3, and 5 years in GSE23143 no data sets were less than 0.7; AUC values of 1, 3, and 5 years in GSE71014 data set were all greater than 0.6) (Figures 4C,D).

### The Risk Score and Tumor-Immune System Interactions Database Analysis

In recent years, immunotherapy for cancer treatment has developed rapidly in AML (Ni et al., 2019). The interaction between tumor and immune system was further analyzed with the help of the TISIDB website. It was found that the expression of genes related to cell receptors was slightly higher in the high-risk group than that in the low-risk group (Supplementary Figure 5A). In addition to differences in *MHC*, *immuno-inhibitor*, *immuno-stimulator*, and *immuno-stimulator* gene in the high-risk group (with generally higher expression), no significant difference was found in other genes in the high-risk group (Supplementary Figure 5B). Finally, the correlation between the expression of immuno-regulator genes and risk score was analyzed, and it was found that *CD96*, *TGFβ1*, *CD160*, *IL-10*, and other genes were highly correlated with the immune score (Supplementary Figure 5C).

### Risk Score-Related Immune Response

**FIGURE 5:**
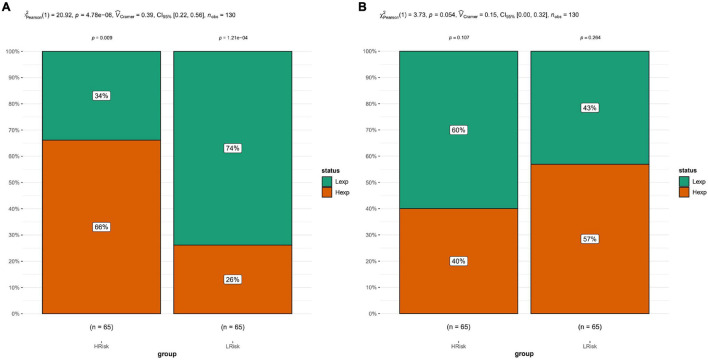
Tide analysis. **(A)** Analysis of immune dysfunction. **(B)** Immune function rejection analysis. The results showed that there were significant differences in immune dysfunction between the high-risk and low-risk groups.

Tumor immunity studies the relationship among body immune function, tumor occurrence, development, and outcome, the mechanism of the immune response to tumors, and the immune effect of tumor cells escaping. Tumor immunotherapy has become an important part of treatment for some cancers, and has provided lasting therapeutic effects for specific patients. Prognostic models emphasize the importance of immunological characteristics in predicting survival to gain a comprehensive understanding of factors that may influence immunotherapy response. It is of great significance for developing precision immune intervention strategies for hematological malignancies (Dufva et al., 2020). Based on the expression profile of AML patients, the TIDE module was used to estimate the immune function and rejection reaction of each patient, and the results revealed that there were significant differences in immune dysfunction between the high-risk and low-risk groups (Figure 5).

### Weighted Gene Co-expression Network Analysis Package Was Used to Identify the Associated Modules

Weighted Gene Co-expression Network Analysis was performed to determine the co-expression network of immune-related genes in the AML cohort. We further constructed the WGCNA network and explored the biomarkers in AML based on the clinical characteristics of high-risk and low-risk groups. The soft threshold β was determined by the function “sft$powerEstimate,” which was set to 7. Next, gene modules were detected based on the TOM matrix, and a total of 15 gene modules were detected [black (*n* = 216), blue (*n* = 804), brown (*n* = 429), cyan (*n* = 82), green (*n* = 312), green-yellow (*n* = 132), gray (*n* = 444), magenta (*n* = 153), pink (*n* = 185), purple (*n* = 146), red (*n* = 303), salmon (*n* = 110), tan (*n* = 131), turquoise (*n* = 1216), and yellow (*n* = 337)]. According to the further analysis of the relationship between modules and traits, the MEblue module was identified to have the highest correlation with the sample category (immune subtype) (cor = 0.43, *p* = 5e-07). Therefore, the MEblue module was selected for the subsequent correlation verification analysis. The modular characteristic genes of the two immune subtypes were analyzed. The characteristic genes in each module showed significant expression among subtypes (Supplementary Figure 6).

## Discussion

Acute myeloid leukemia, as a common malignancy, has seen an increasing mortality rate in recent years. Myeloid leukemia cells are inherently in a state of oxidative stress due to impaired ROS homeostasis, which is a common feature of several hematological malignancies (Trombetti et al., 2021). ROS, as a product of normal cell metabolism, could regulate cell survival, proliferation, apoptosis, and cell cycle, as well as cell homeostasis. Most ROS are produced in cells by the mitochondrial respiratory chain. Oxidative stress is affected by some pathological factors. The free radicals and ROS produced by oxidation of the antioxidant defense system imbalance the cell environment, influence pathways related to cell survival, proliferation, and apoptosis, promote tumor cell survival, induce cell proliferation, protect cells from apoptosis, and promote the occurrence of various cancers. Recent studies have shown that the REDOX disorder caused by ROS promotes the development of leukemia and is closely related to the treatment and prognosis of leukemia. Moreover, oxidative stress is also tightly linked with the recurrence of AML. AML relapse is a tumor state that is susceptible to antioxidant defense, oxidative damage, and fundamental genetic changes. Moreover, the correlation between oxidative stress and AML recurrence rate has potential prognostic significance (Zhou et al., 2010). The application of tumor risk models is increasingly being recognized in current research. Selecting the oxidative stress-related biomarkers in the early progression of AML and establishing a risk prediction model for AML can better manage leukemia and improve the prognosis of leukemia. Most previous therapies only target tumor cells, and rarely focus on the tumor microenvironment. Therefore, an in-depth study on the leukemia microenvironment may help to further understand the pathogenesis of leukemia and to seek new targets for microenvironment therapy (Wang et al., 2020).

Currently, among the prognosis models of leukemia, there is only one GEO external validation for the leukemia model. In this study, two GEO data sets were used for external validation, and the model has good validation efficiency. We examined the prognostic value of the model using multi-omics. First, the relationship between risk scores and tumor immuno-invasion was analyzed. The results showed that there was a significant correlation between risk score and immune cells. Immuno-invasion is closely associated with tumor immunotherapy; therefore, the risk score may be useful in predicting immunotherapy. Next, IC50 (half-inhibitory concentration) was used to investigate the differences in sensitivity to common chemotherapeutic agents between the high-risk and low-risk groups. The results showed that risk scores significantly affected patients’ susceptibility to Bexarotene, Bortezomib, Bryostatin 1, Dasatinib, Metformin, and Paclitaxel. The mutations in the genes of patients in the high-risk and low-risk groups were shown by the mutation map.

Bexarotene, defined as a specific Retinoid X Receptor (RXRS) agonist, has been approved by the FDA for the clinical treatment of cutaneous T cell lymphoma (CTCL). Bexarotene is an effective and rational method for chemoprophylaxis and the treatment of a variety of cancers. Combined with other cellular agents or targeted agents, it may significantly enhance the efficacy and reduce the toxicity due to its non-overlapping side effects. The use of bexarotene in non-M3 AML has gained attention (DiNardo et al., 2008; McNamara and Miller, 2008; Tsai et al., 2008; Shen et al., 2018). Bortezomib enhances γδT cell-mediated AML and T-ALL cell killing in part by increasing NKG2D ligand-receptor interactions. Bortezomib combined with artesunate has obvious synergistic effects on the proliferation, apoptosis, and autophagy of MV4-11 cell lines, which may be associated with the expression of Bcl-2 family proteins. The treatment of human leukemia HL60 xenografts using Bortezomib and ATRA not only increased the differentiation, but also inhibited tumor growth (Ying et al., 2013; Hu et al., 2019; Moore et al., 2020; Story et al., 2021). Bryostatin-1 notably inhibited cell proliferation and induced NB4 cell differentiation into monocytes/macrophages. The combination of bryostatin-1 and 1alpha, 25(OH)2D3 affects NB4 cell differentiation and proliferation. Therefore, combination therapy may be another potential treatment option for AML patients (Song and Norman, 1999). As has been evidenced previously, bryostatin-1 can enhance the pro-apoptotic effect of Treosulfan (Schmidmaier et al., 2004). Dasatinib, as a potent BCR-ABL inhibitor, is resistant/intolerant to imatinib and has been studied in combination with multiple chemotherapy regimens in AML patients aged 13–15 years (Foà et al., 2011; Kivioja et al., 2019; Welch, 2020). Chemotherapy combined with Dasatinib has a good efficacy in both young and old AML patients with or without mutation or overexpressing KIT (Marcucci et al., 2020). Resistance remains a major barrier for AML patients; therefore, more effective and less toxic therapies are urgently needed. Metformin, a classic hypoglycemic agent for diabetes mellitus, has recently been found to have anti-tumor activity by inhibiting mTOR in various tumors. Metformin synergically sensitized AML cells to Ara-C via inhibiting the mTORC1/p70S6K pathway, exhibiting significantly synergistic anti-tumor effects (Visnjic et al., 2019; Yuan et al., 2020). Metformin also plays an anti-leukemia role by activating p-AMPK and cooperatively sensitizes FLT3-mutated AML to sorafenib (Wang et al., 2015). Metformin regulates the treatment of chemically resistant AML cells by chemical metabolic pathways, which can be enhanced by the combination of metformin with cytarabine and venetoclax (Vitkevičienė et al., 2019). Clinical cases have suggested that T-AML may occur after paclitaxel treatment, which underscores the importance of assessing leukemia risk factors before starting paclitaxel treatment (Yeasmin et al., 2008; Saito et al., 2009). On the other hand, paclitaxel inhibits the growth of HL-60 cells *in vitro* by inducing cell cycle arrest and apoptosis (Wan et al., 2004).

Subsequently, the specific signaling pathways involved in the high-low risk correlation model were analyzed through GSEA analysis. Both GO and KEGG results showed that patients in the high-risk group showed association with some common tumor-related signaling pathways. This suggested that the disturbance of these signaling pathways in the high-risk group may be a possible mechanism affecting the prognosis of AML patients. To verify the accuracy of the model, we downloaded two data sets of GO to discuss the robustness of the model. It was found that the two external data were significant, which indicated that this model had a strong predictive power for the prognosis of patients.

Finally, the relationship among oxidative stress genes, immuno-regulatory factors, and the corresponding immune responses were studied by immuno-analysis and immunotherapy prediction. The results showed that *CD96*, *TGFβ1*, *CD160*, *IL-10*, and other genes were highly correlated with the expression of immuno-regulator genes and risk score. In terms of the relevant immune response, the TIDE module was used to estimate the immune function and rejection reaction of each patient, and the results revealed that there were significant differences in immune dysfunction between the high-risk and low-risk groups. *CD96*, a transmembrane glycoprotein, is reported to be expressed only on T cells and NK cells. The expression of *CD96* can distinguish AML LSCs from normal HSC. *CD96* is a specific marker of leukemia stem cells in human AML, which is a good candidate target for targeting LSC (Hosen et al., 2007; Wang et al., 2013; Ding et al., 2017; Jiang et al., 2017). The decrease of *TGFβ1* expression in AML patients is closely related to the poor prognosis of AML patients, which can provide a reference for improving the clinical efficacy of AML patients (Tamai et al., 2017; Chen W. T. et al., 2020). Natural killer (NK) cells are congenital cytotoxic lymphoid cells (ILC) that are involved in killing the infected cells and tumor cells. *CD160* levels of bone marrow NK cells were reduced in AML patients, but the survival rate was higher in the high-*CD160* group (Crinier et al., 2021). The cytolytic function of NK cells is partially mediated by *CD160* molecules. Reduced *CD160* expression on NK cells in patients with various hematological malignancies suggests that down-regulation of *CD160* may be a novel mechanism of tumor immune escape (Wang et al., 2018). A previous article has shown that *IL-10* is related to AML cell survival, proliferation, and differentiation (Yan et al., 2019). The association between *IL-10* gene polymorphism and human AML has been verified in many countries (Yao et al., 2013; Rashed et al., 2018; Sharif et al., 2019).

Overall, this study is based on the prognostic genes related to oxidative stress to explore the multi-omics of AML, which has certain guiding effects on the prognosis of AML. However, our study still has some limitations. First, the total number of AML patients in our study was limited, and larger data sets are urgently needed in future studies to further validate our predictive model. Secondly, only target data from TCGA public database through biological algorithms were selected, which has some limitations. Finally, biological experiments and clinical trials are needed to verify and support the results of this study.

## Conclusion

In summary, the multi-omics mechanism of the prognostic risk model for leukemia has been explored in its drug sensitivity, signaling pathways, and immune infiltration. We have established a reliable prognostic model, which can guide the prognosis of AML more accurately, and will provide a new basis for disease management. Cancer prediction models have successfully implemented early detection and intervention plans for several solid tumors. However, screening tests are not suitable for the subclinical stage of most hematological malignancies. Our study provides a proof of concept for the feasibility of early detection of high-risk healthy individuals.

## Data Availability Statement

The original contributions presented in the study are included in the article/Supplementary Material, further inquiries can be directed to the corresponding author/s.

## Author Contributions

LZ finished the study design. NZ finished the experimental studies. CD took part in data analysis and finished the manuscript editing. All authors read and approved the final manuscript.

## Conflict of Interest

The authors declare that the research was conducted in the absence of any commercial or financial relationships that could be construed as a potential conflict of interest.

## Publisher’s Note

All claims expressed in this article are solely those of the authors and do not necessarily represent those of their affiliated organizations, or those of the publisher, the editors and the reviewers. Any product that may be evaluated in this article, or claim that may be made by its manufacturer, is not guaranteed or endorsed by the publisher.

## Footnotes

1
https://portal.gdc.cancer.gov/

2
http://amigo.geneontology.org/amigo

3
www.cancerRxgene.org

4
http://www.broadinstitute.org/gsea

5
http://cis.hku.hk/TISIDB/index.php

6
http://tide.dfci.harvard.edu/

## Supplementary Material

Supplementary Figure 1 **(A–L)** Display of clustering results with different *K* values. When the cluster number *K* = 3, AML patients could be allocated into three subgroups. **(A)** The color legend of the matrix heat map. **(B–I)** The matrix heat map, when *k* = 2, 3, 4, 5, 6, 7, 8, 9, the rows and columns of the matrix were all samples, and the values of the consistency matrix were set from 0 (impossible to aggregate) to 1 (always clustered together), and were represented in white to dark blue; the consistency matrix was arranged according to the consistency classification (the tree diagram above the heat map); the long bar between tree map and the heat map was the category. **(J)** The uniform cumulative distribution function (CDF) diagram that displayed the CDF with different *k* values, which was used to determine the approximate maximum value of CDF when k took a value, and at this time, the cluster analysis result was the most reliable. That is, consider the small *k* value of the CDF descending slope. **(K)** Delta Area Plots shows the relative change of the area under the CDF curve between *k* and *k*-1; when *k* = 2 (there is no *k* = 1), the first point represents the total area under the CDF curve, rather than the relative change value of the area. **(L)** Tracking Plot. The black stripe at the bottom of this figure represented the sample; the classification of the sample was shown when k took different values, and different color patches represented different classifications. Samples with often changed color classification before and after different *k* values represented unstable classification, and more samples with unstable classification were indicative of more unstable classification under the *k* value.

Supplementary Figure 2 Prediction analysis of survival time between different groups obtained by clustering at *K* = 3. Results showed that there were significant differences in survival between the three groups, with subgroup 1 showing the worst prognosis and subgroup 3 showing the best prognosis.

Supplementary Figure 3 A correlation analysis of immune factors and risk scores. **(A)** Risk score was significantly positively correlated with monocytes, macrophages M2, and activated CD4 memory T cells, and negatively correlated with eosinophils, T cells gamma delta, and mast cells resting. **(B)** Sensitivity analysis of samples from the high-risk and low-risk groups to immuno-drugs. They were significant between groups by the rank sum test. Risk score notably affected patients’ susceptibility to Bexarotene, Bortezomib, Bryostatin 1, Dasatinib, Metformin, and Paclitaxel. **(C)** Analysis of gene mutation frequency in the high-risk and low-risk groups. At the bottom of the image, the yellow fragment corresponds to the high-risk group, the blue bars represent the low-risk group, and the colored bars represent the different types of mutations.

Supplementary Figure 4 GSEA enrichment analysis. **(A)** Showed the significant enrichment of some pathways in GO enrichment analysis. GO results showed that patients in the high-risk group mainly enriched TUMOR NECROSIS FACTOR MEDIATED SIGNALING PATHWAY, INTERFERON GAMMA PRODUCTION, and other SIGNALING pathways. **(B)** Showed the significant enrichment of some pathways in KEGG enrichment analysis. Patients in the high-risk group were mainly enriched in the MEDIATED CYTOTOXICITY of NATURAL KILLER CELL, Chemokine SIGNALING PATHWAY, and other SIGNALING pathways. These results suggested that the disturbance of these signaling pathways in patients (high-risk and low-risk) may affect the prognosis of AML patients.

Supplementary Figure 5 TISIDB analysis. **(A)** The expression profile of cell receptor-related genes in the high-risk and low-risk groups. The results showed that the expression profile of cell receptor-related genes in the high-risk group was slightly higher than that in the low-risk group. **(B)** Expression profiles of immuno-regulatory and chemokine-related genes in the high-risk and low-risk groups. In addition to differences in *MHC*, *Immuno-inhibitor*, *Immuno-stimulator*, and *Immuno-stimulator* gene in the high-risk group (the expression was generally higher in the high-risk group), no significant difference was found in other genes in the high-risk group. **(C)** Correlation analysis of the expression of immuno-regulatory sub-genes and risk scores. The results showed that *CD96*, *TGFβ1*, *CD160*, *IL10*, and other genes were highly correlated with the immune score.

Supplementary Figure 6 Construction of immune gene WGCNA co-expression network. **(A)** Sample clustering (excluding some abnormal samples). **(B)** Select the best soft thresholding power. **(C)** Construct the co-expression matrix (module recognition). **(D)** Similar module clustering. **(E)** Module visualization. **(F)** The correlation between module and subtype. **(G)** Correlation analysis of module gene and subtype. **(H)** Functional analysis of modular genes.
