# Supplementary figures and images for: The Multi-Omic Prognostic Model of Oxidative Stress-Related Genes in Acute Myeloid Leukemia
Source: Front Genet. 2021 Sep 30;12:722064. doi: 10.3389/fgene.2021.722064 (PMC8514868; doi:10.3389/fgene.2021.722064)

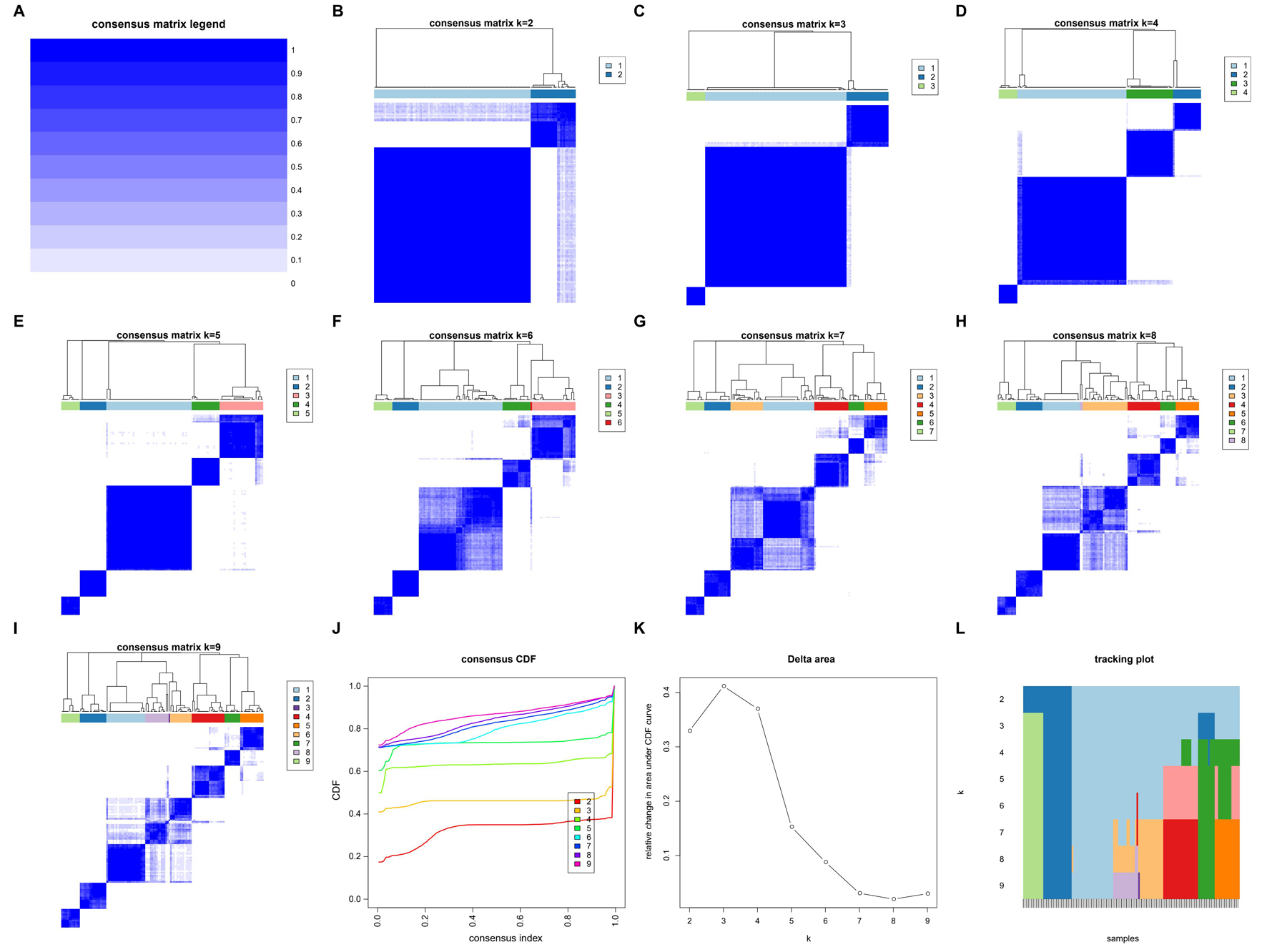

Supplement: Supplementary Figure 1 — (A–L) Display of clustering results with different K values. When the cluster number K = 3, AML patients could be allocated into three subgroups. (A) The color legend of the matrix heat map. (B–I) The matrix heat map, when k = 2, 3, 4, 5, 6, 7, 8, 9, the rows and columns of the matrix were all samples, and the values of the consistency matrix were set from 0 (impossible to aggregate) to 1 (always clustered together), and were represented in white to dark blue; the consistency matrix was arranged according to the consistency classification (the tree diagram above the heat map); the long bar between tree map and the heat map was the category. (J) The uniform cumulative distribution function (CDF) diagram that displayed the CDF with different k values, which was used to determine the approximate maximum value of CDF when k took a value, and at this time, the cluster analysis result was the most reliable. That is, consider the small k value of the CDF descending slope. (K) Delta Area Plots shows the relative change of the area under the CDF curve between k and k-1; when k = 2 (there is no k = 1), the first point represents the total area under the CDF curve, rather than the relative change value of the area. (L) Tracking Plot. The black stripe at the bottom of this figure represented the sample; the classification of the sample was shown when k took different values, and different color patches represented different classifications. Samples with often changed color classification before and after different k values represented unstable classification, and more samples with unstable classification were indicative of more unstable classification under the k value. [file Image_1.JPEG]

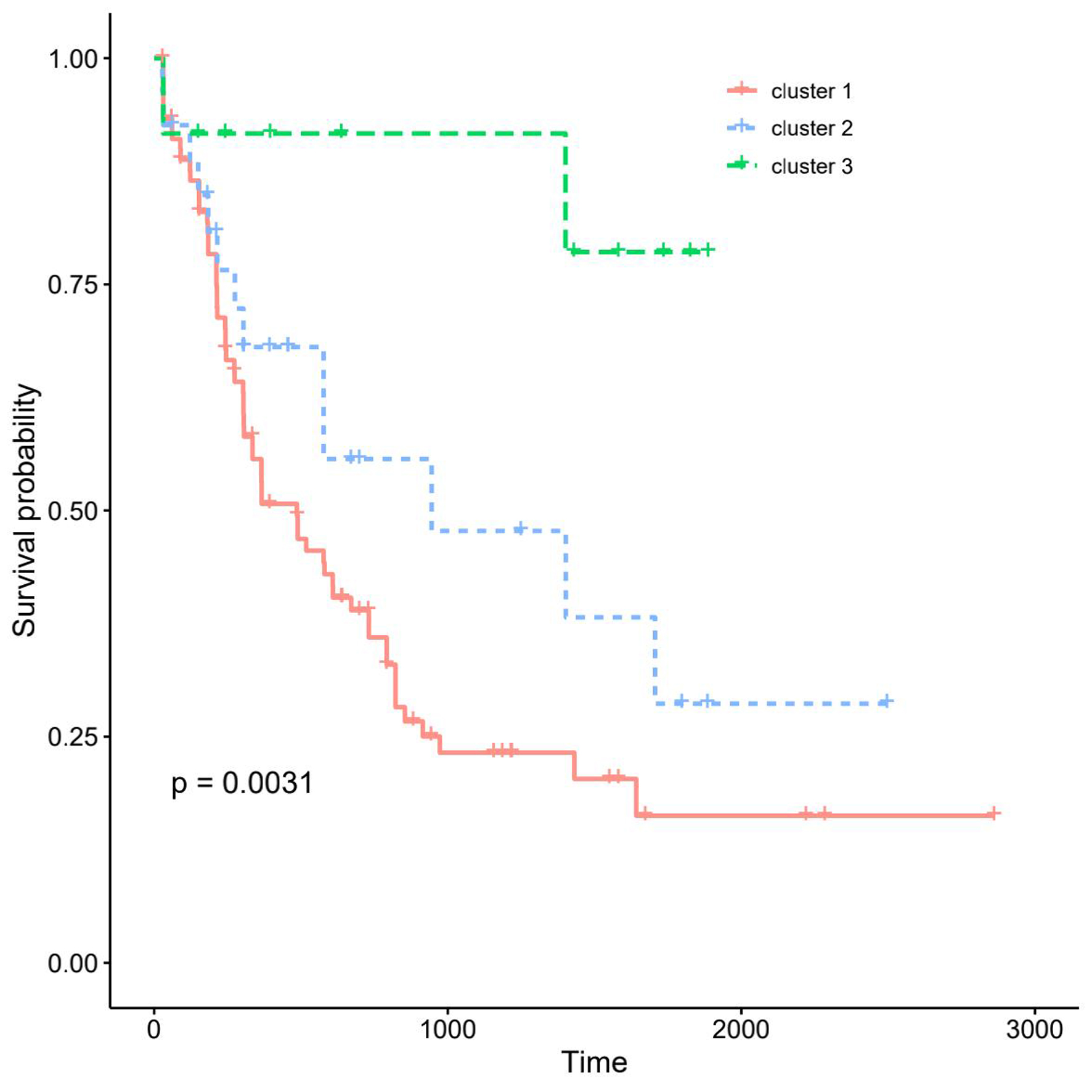

Supplement: Supplementary Figure 2 — Prediction analysis of survival time between different groups obtained by clustering at K = 3. Results showed that there were significant differences in survival between the three groups, with subgroup 1 showing the worst prognosis and subgroup 3 showing the best prognosis. [file Image_2.JPEG]

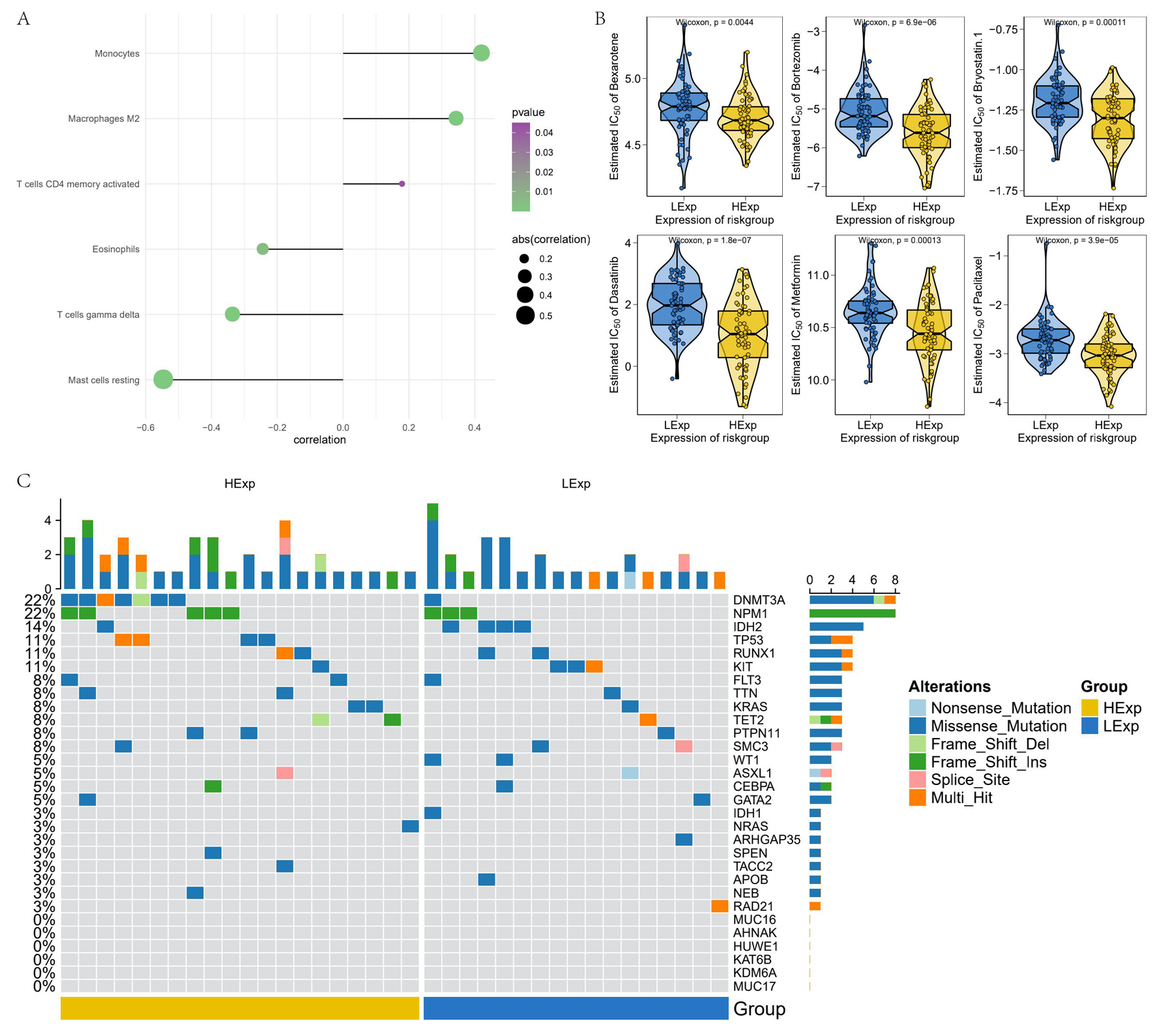

Supplement: Supplementary Figure 3 — A correlation analysis of immune factors and risk scores. (A) Risk score was significantly positively correlated with monocytes, macrophages M2, and activated CD4 memory T cells, and negatively correlated with eosinophils, T cells gamma delta, and mast cells resting. (B) Sensitivity analysis of samples from the high-risk and low-risk groups to immuno-drugs. They were significant between groups by the rank sum test. Risk score notably affected patients’ susceptibility to Bexarotene, Bortezomib, Bryostatin 1, Dasatinib, Metformin, and Paclitaxel. (C) Analysis of gene mutation frequency in the high-risk and low-risk groups. At the bottom of the image, the yellow fragment corresponds to the high-risk group, the blue bars represent the low-risk group, and the colored bars represent the different types of mutations. [file Image_3.JPEG]

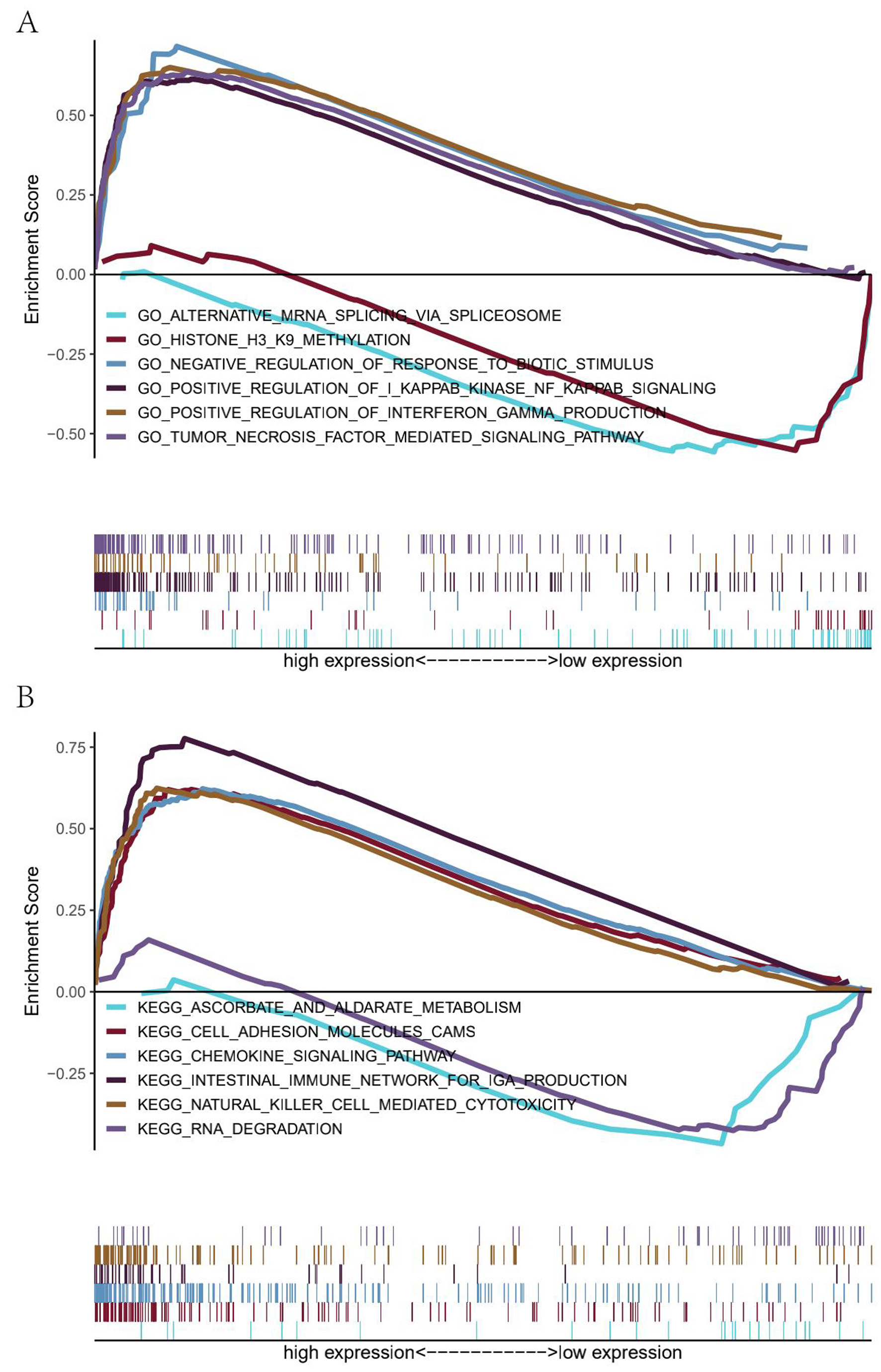

Supplement: Supplementary Figure 4 — GSEA enrichment analysis. (A) Showed the significant enrichment of some pathways in GO enrichment analysis. GO results showed that patients in the high-risk group mainly enriched TUMOR NECROSIS FACTOR MEDIATED SIGNALING PATHWAY, INTERFERON GAMMA PRODUCTION, and other SIGNALING pathways. (B) Showed the significant enrichment of some pathways in KEGG enrichment analysis. Patients in the high-risk group were mainly enriched in the MEDIATED CYTOTOXICITY of NATURAL KILLER CELL, Chemokine SIGNALING PATHWAY, and other SIGNALING pathways. These results suggested that the disturbance of these signaling pathways in patients (high-risk and low-risk) may affect the prognosis of AML patients. [file Image_4.JPEG]

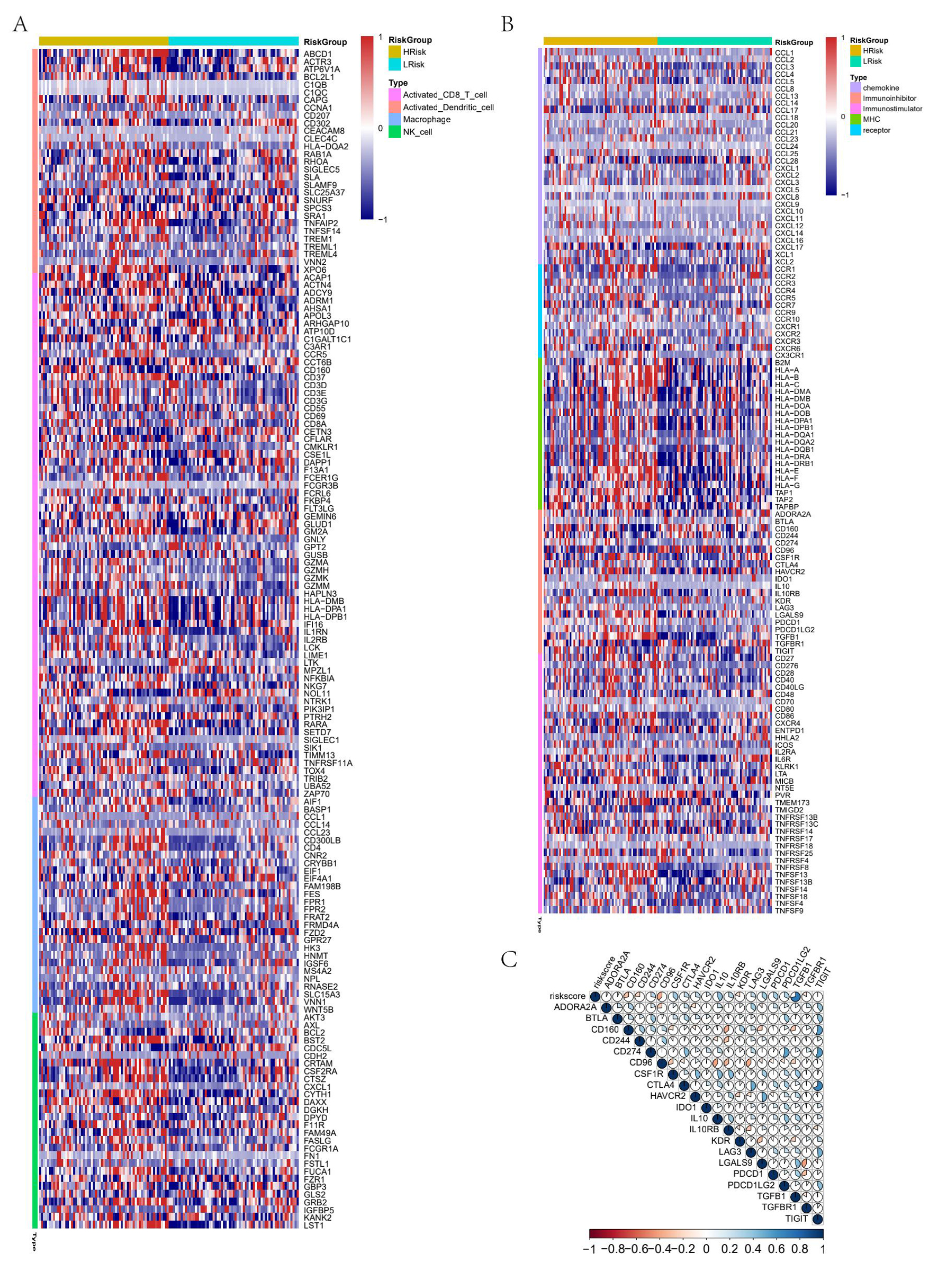

Supplement: Supplementary Figure 5 — TISIDB analysis. (A) The expression profile of cell receptor-related genes in the high-risk and low-risk groups. The results showed that the expression profile of cell receptor-related genes in the high-risk group was slightly higher than that in the low-risk group. (B) Expression profiles of immuno-regulatory and chemokine-related genes in the high-risk and low-risk groups. In addition to differences in MHC, Immuno-inhibitor, Immuno-stimulator, and Immuno-stimulator gene in the high-risk group (the expression was generally higher in the high-risk group), no significant difference was found in other genes in the high-risk group. (C) Correlation analysis of the expression of immuno-regulatory sub-genes and risk scores. The results showed that CD96, TGFβ1, CD160, IL10, and other genes were highly correlated with the immune score. [file Image_5.JPEG]

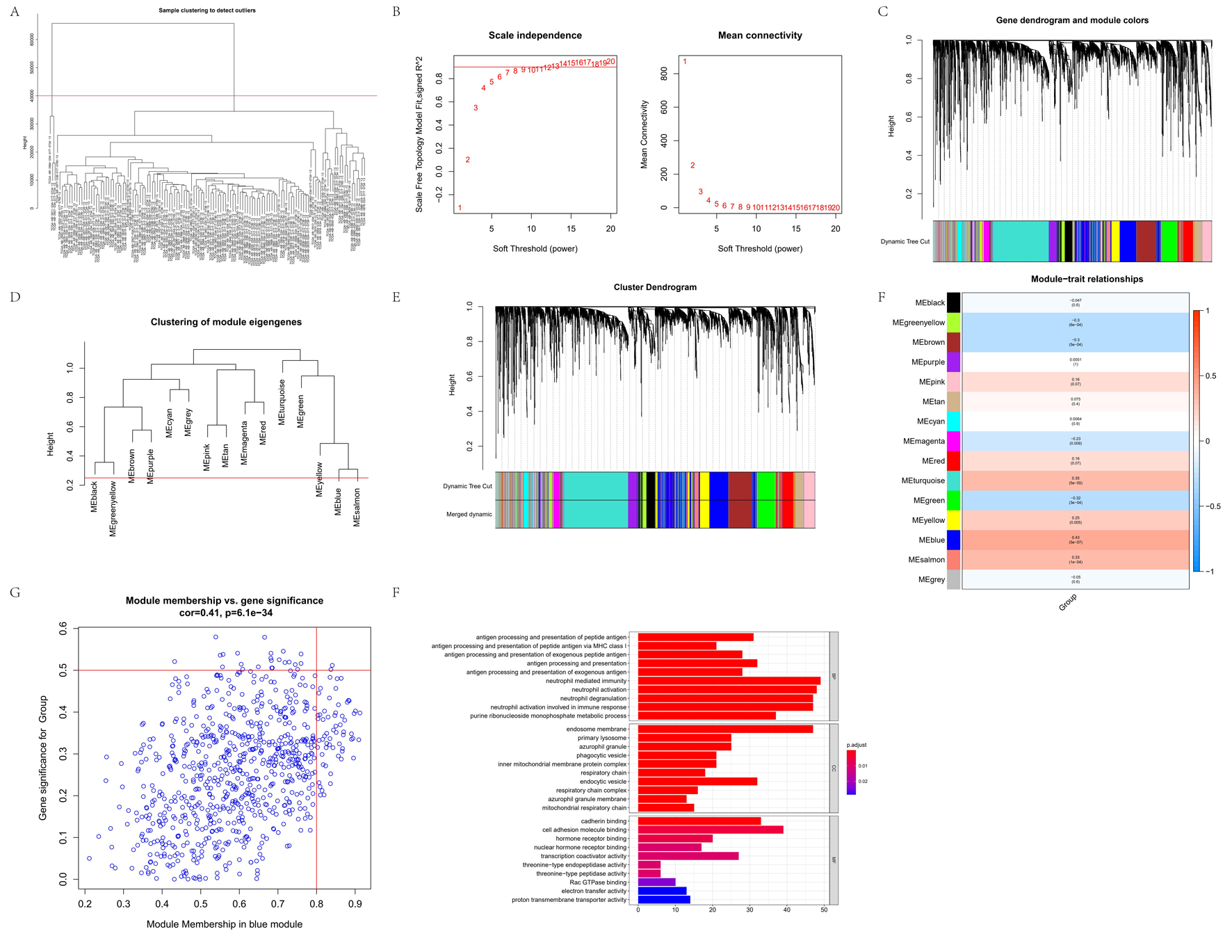

Supplement: Supplementary Figure 6 — Construction of immune gene WGCNA co-expression network. (A) Sample clustering (excluding some abnormal samples). (B) Select the best soft thresholding power. (C) Construct the co-expression matrix (module recognition). (D) Similar module clustering. (E) Module visualization. (F) The correlation between module and subtype. (G) Correlation analysis of module gene and subtype. (H) Functional analysis of modular genes. [file Image_6.JPEG]
